# Supplementary material for: Evaluation of Fetal Cardiac Geometry and Contractility in Gestational Diabetes Mellitus by Two-Dimensional Speckle-Tracking Technology
Source: Diagnostics (Basel). 2022 Aug 24;12(9):2053. doi: 10.3390/diagnostics12092053 (PMC9497478; doi:10.3390/diagnostics12092053)
Supplement: Supplementary file 1 [file diagnostics-12-02053-s001.zip › Supplements tables.pdf]

diagnostics

MDPI

Table S1. Comparison of fetal cardiac indices between the Control group and the GD group.

Parameter	Measuring unit	Control (*n* = 33)*	GD (*n* = 33)*	*p*
**4CV-GSI**	Value	1.21 ± 0.06	1.23 ± 0.08	0.461
	Z-score	-0.15 ± 0.71	0.01 ± 0.94	0.465
**LV-GLS**	%	-24.33 ± 4.27	-22.16 ± 5.8	0.088
**RV-GLS**	%	-23.39 ± 3.59	-21.42 ± 5.8	0.102
**LV-FAC**	%	47.17 ± 6.24	45.26 ± 8.38	0.295
	Z-AC	0.33 ± 1.24	-0.07 ± 1.83	0.294
	Z-EFW	0.3 ± 1.45	-0.15 ± 2.65	0.396
	Z-GA	0.33 ± 1.23	-0.09 ± 1.83	0.267
**RV-FAC**	%	40.33 ± 5.42	33.27 ± 9.66	<0.001
	Z-AC	0.06 ± 1.03	-1.26 ± 1.86	<0.001
	Z-EFW	0.04 ± 1.03	-1.29 ± 1.84	<0.001
	Z-GA	0.01 ± 1.03	-1.32 ± 1.84	<0.001
**RV-ES-Area**	cm^2	1.56 ± 0.44	1.8 ± 0.64	0.035
**LV-EF**	%	61.64 ± 7.76	59.8 ± 9.83	0.401
	Z-AC	-0.06 ± 1.19	-0.38 ± 1.58	0.348
	Z-EFW	-0.07 ± 1.18	-0.39 ± 1.58	0.363
	Z-GA	-0.08 ± 1.19	-0.39 ± 1.6	0.381
**LV-SV**	ml	1.6 ± 0.53	1.53 ± 0.43	0.644
	Z-AC	0.07 ± 0.92	-0.24 ± 0.87	0.143
	Z-EFW	0.08 ± 1.01	-0.15 ± 0.97	0.317
	Z-GA	0.14 ± 0.94	0.01 ± 0.97	0.545
**LV-SV/KG**	ml/kg	0.71 ± 0.18	0.66 ± 0.19	0.228
	Z-AC	0.15 ± 0.93	-0.08 ± 0.94	0.308
	Z-EFW	0.12 ± 0.96	-0.11 ± 0.94	0.303
	Z-GA	0.12 ± 0.92	-0.15 ± 0.95	0.236
**LV-CO**	ml/min	224.51 ± 76.69	209.56 ± 58.27	0.621
	Z-AC	0 ± 0.95	-0.4 ± 0.84	0.069
	Z-EFW	0.02 ± 1.04	-0.32 ± 0.94	0.162
	Z-GA	0.02 ± 0.93	-0.19 ± 0.91	0.327
**LV-CO/KG**	ml/min/kg	100.46 ± 25.79	90.35 ± 25.46	0.122
	Z-AC	0.09 ± 0.94	-0.25 ± 0.91	0.152
	Z-EFW	0.09 ± 0.96	-0.25 ± 0.9	0.182
	Z-GA	0.06 ± 0.94	-0.3 ± 0.91	0.152

* Data are given as mean ± SD. GD, gestational diabetes mellitus; *p*, *p*-value; 4CV-GSI, four-chamber view global sphericity index; GLS, global longitudinal strain; FAC, fractional area change; Z-AC, Z-score adjusted to the abdominal circumference; Z-EFW, Z-score adjusted to the estimated fetal weight; Z-GA, Z-score adjusted to the gestational age; ES, end-systolic; EF, ejection fraction; SV, stroke volume; CO, cardiac output.

Table S2. Comparison of the 24-segment End-Diastolic Diameter between the Control group and the GD group.

24-segments				1	2	3	4	5	6	7	8	9	10	11	12	13	14	15	16	17	18	19	20	21	22	23	24
EDD (mm)	LV	Control	Mean	13.19	13.45	13.71	13.89	13.92	13.77	13.47	13.08	12.68	12.3	11.93	11.57	11.18	10.76	10.32	9.9	9.54	9.22	8.85	8.28	7.35	5.96	4.18	2.15
			SD	1.94	1.82	1.76	1.75	1.75	1.73	1.7	1.66	1.63	1.6	1.56	1.52	1.49	1.46	1.43	1.43	1.45	1.5	1.55	1.57	1.5	1.28	0.93	0.48
		GD	Mean	13.88	13.96	14.02	14.03	13.94	13.7	13.36	12.97	12.56	12.18	11.81	11.43	11.04	10.62	10.2	9.8	9.45	9.16	8.82	8.29	7.39	6.02	4.24	2.19
			SD	2.03	1.87	1.75	1.68	1.63	1.6	1.57	1.56	1.56	1.57	1.57	1.58	1.59	1.6	1.6	1.6	1.6	1.6	1.6	1.57	1.47	1.24	0.89	0.46
			p	0.160	0.274	0.476	0.740	0.918	0.827	0.744	0.667	0.682	0.758	0.768	0.724	0.682	0.581	0.542	0.547	0.573	0.658	0.788	0.985	0.710	0.644	0.603	0.573
	RV	Control	Mean	14.05	14.13	14.21	14.26	14.25	14.15	13.98	13.73	13.42	13.05	12.64	12.19	11.72	11.24	10.76	10.29	9.84	9.41	8.91	8.23	7.23	5.83	4.08	2.1
			SD	2.14	2.01	1.92	1.88	1.86	1.83	1.81	1.79	1.77	1.74	1.71	1.69	1.67	1.65	1.62	1.6	1.59	1.59	1.61	1.61	1.53	1.3	0.94	0.49
		GD	Mean	14.36	14.34	14.32	14.28	14.2	14.06	13.87	13.63	13.36	13.04	12.69	12.3	11.88	11.42	10.92	10.41	9.89	9.34	8.72	7.93	6.88	5.49	3.82	1.95
			SD	2.92	2.8	2.72	2.66	2.63	2.61	2.62	2.63	2.65	2.64	2.63	2.61	2.58	2.54	2.49	2.42	2.33	2.21	2.08	1.92	1.72	1.41	1	0.52
			p	0.739	0.974	0.827	0.686	0.581	0.501	0.415	0.445	0.445	0.538	0.505	0.534	0.649	0.705	0.768	0.705	0.724	0.581	0.581	0.449	0.397	0.373	0.423	0.442
EDD (Z-AC)	LV	Control	Mean	0.15	0.19	0.31	0.41	0.44	0.38	0.26	0.1	-0.06	-0.2	-0.33	-0.44	-0.53	-0.62	-0.7	-0.78	-0.88	-0.98	-1.07	-1.12	-1.12	-1.09	-1.08	-1.07
			SD	0.92	0.81	0.76	0.78	0.79	0.79	0.77	0.77	0.76	0.76	0.76	0.76	0.75	0.74	0.73	0.75	0.8	0.88	0.97	1.04	1.05	1.04	1.03	1.03
		GD	Mean	0.36	0.27	0.28	0.29	0.25	0.16	0.03	-0.12	-0.28	-0.41	-0.53	-0.63	-0.71	-0.78	-0.85	-0.91	-0.99	-1.07	-1.13	-1.15	-1.1	-1.05	-1.02	-1.01
			SD	0.96	0.82	0.75	0.71	0.69	0.69	0.71	0.74	0.79	0.82	0.87	0.89	0.9	0.91	0.91	0.93	0.96	1.01	1.06	1.09	1.08	1.05	1.04	1.03
			p	0.369	0.685	0.881	0.511	0.306	0.221	0.202	0.220	0.259	0.296	0.419	0.469	0.383	0.343	0.290	0.352	0.383	0.445	0.654	0.852	0.857	0.710	0.644	0.612
	RV	Control	Mean	0.85	0.82	0.79	0.73	0.64	0.52	0.4	0.26	0.13	0	-0.11	-0.22	-0.29	-0.36	-0.38	-0.37	-0.34	-0.27	-0.18	-0.07	0	0.05	0.08	0.09
			SD	1.05	1.04	1.02	1.04	1.05	1.07	1.07	1.06	1.05	1.04	1.02	1.01	1.01	1	0.99	1	1.05	1.16	1.29	1.36	1.38	1.37	1.36	1.36
		GD	Mean	0.74	0.64	0.52	0.39	0.26	0.12	0	-0.11	-0.22	-0.3	-0.38	-0.43	-0.48	-0.51	-0.53	-0.54	-0.54	-0.55	-0.54	-0.5	-0.45	-0.41	-0.39	-0.38
			SD	1.56	1.55	1.51	1.48	1.47	1.5	1.51	1.5	1.49	1.47	1.45	1.41	1.38	1.38	1.36	1.36	1.4	1.49	1.59	1.58	1.53	1.48	1.45	1.43
			p	0.725	0.571	0.395	0.293	0.229	0.220	0.215	0.233	0.273	0.327	0.400	0.478	0.548	0.610	0.612	0.574	0.499	0.402	0.311	0.242	0.205	0.183	0.175	0.169
EDD (Z-EFW)	LV	Control	Mean	0.04	0.18	0.31	0.42	0.45	0.4	0.28	0.12	-0.05	-0.21	-0.34	-0.42	-0.51	-0.63	-0.65	-0.73	-0.82	-0.93	-1.02	-1.08	-1.07	-1.06	-1.05	-1.04
			SD	0.81	0.78	0.77	0.79	0.81	0.82	0.79	0.79	0.8	0.8	0.8	0.74	0.73	0.77	0.71	0.72	0.76	0.83	0.91	0.97	0.97	0.96	0.96	0.95
		GD	Mean	0.3	0.35	0.38	0.39	0.35	0.25	0.11	-0.04	-0.21	-0.36	-0.49	-0.57	-0.66	-0.75	-0.78	-0.84	-0.92	-1	-1.07	-1.09	-1.06	-1.02	-0.99	-0.98
			SD	0.84	0.8	0.75	0.72	0.71	0.72	0.73	0.78	0.83	0.87	0.91	0.91	0.91	0.92	0.94	0.95	0.97	1	1.05	1.06	1.04	1.02	1	1
			p	0.204	0.392	0.728	0.873	0.591	0.455	0.379	0.461	0.440	0.467	0.577	0.493	0.497	0.542	0.317	0.336	0.376	0.457	0.551	0.758	0.954	0.778	0.715	0.710
	RV	Control	Mean	0.87	0.87	0.84	0.76	0.7	0.61	0.5	0.39	0.25	0.11	-0.01	-0.12	-0.21	-0.27	-0.29	-0.28	-0.27	-0.2	-0.13	-0.06	0.01	0.05	0.08	0.09
			SD	1.01	0.99	1	1.03	1.06	1.09	1.11	1.13	1.12	1.11	1.09	1.08	1.07	1.06	1.04	1.05	1.09	1.17	1.26	1.32	1.35	1.35	1.35	1.35
		GD	Mean	0.9	0.82	0.7	0.57	0.45	0.32	0.19	0.08	-0.02	-0.12	-0.2	-0.27	-0.33	-0.37	-0.39	-0.41	-0.44	-0.44	-0.45	-0.44	-0.4	-0.38	-0.36	-0.35
			SD	1.5	1.49	1.45	1.44	1.45	1.46	1.5	1.51	1.52	1.51	1.48	1.45	1.42	1.38	1.34	1.33	1.4	1.47	1.55	1.58	1.55	1.51	1.49	1.47
			p	0.936	0.870	0.667	0.529	0.426	0.368	0.355	0.350	0.394	0.459	0.542	0.631	0.704	0.739	0.727	0.673	0.600	0.468	0.366	0.300	0.248	0.221	0.207	0.201
EDD (Z-GA)	LV	Control	Mean	0.2	0.32	0.43	0.52	0.54	0.49	0.39	0.26	0.12	0	-0.1	-0.19	-0.28	-0.36	-0.44	-0.52	-0.61	-0.7	-0.78	-0.84	-0.86	-0.86	-0.86	-0.86
			SD	0.82	0.77	0.74	0.73	0.73	0.73	0.72	0.72	0.73	0.73	0.74	0.74	0.75	0.75	0.75	0.78	0.83	0.9	0.98	1.04	1.05	1.05	1.04	1.04
		GD	Mean	0.51	0.55	0.58	0.58	0.54	0.47	0.35	0.22	0.08	-0.03	-0.14	-0.25	-0.34	-0.42	-0.49	-0.56	-0.63	-0.71	-0.77	-0.8	-0.79	-0.77	-0.76	-0.75
			SD	0.83	0.77	0.72	0.69	0.67	0.67	0.68	0.7	0.73	0.77	0.8	0.82	0.84	0.86	0.87	0.89	0.93	0.97	1.02	1.04	1.03	1.02	1.01	1
			p	0.132	0.238	0.440	0.581	0.798	0.869	0.778	0.672	0.724	0.858	0.827	0.748	0.729	0.654	0.644	0.663	0.715	0.923	0.995	0.773	0.581	0.534	0.493	0.445
	RV	Control	Mean	0.72	0.71	0.69	0.66	0.61	0.54	0.45	0.35	0.25	0.15	0.06	0.01	-0.04	-0.08	-0.1	-0.1	-0.1	-0.05	0.02	0.09	0.15	0.18	0.2	0.21
			SD	0.95	0.91	0.88	0.87	0.87	0.86	0.86	0.86	0.85	0.84	0.83	0.84	0.84	0.84	0.85	0.86	0.89	0.96	1.06	1.15	1.22	1.25	1.26	1.27
		GD	Mean	0.85	0.8	0.73	0.64	0.55	0.46	0.36	0.27	0.19	0.11	0.05	0.04	0	-0.02	-0.04	-0.06	-0.1	-0.1	-0.11	-0.12	-0.12	-0.13	-0.13	-0.13
			SD	1.3	1.28	1.25	1.23	1.21	1.21	1.22	1.23	1.24	1.23	1.22	1.24	1.24	1.24	1.25	1.27	1.27	1.32	1.37	1.39	1.39	1.38	1.37	1.36
			p	0.622	0.749	0.897	0.958	0.842	0.767	0.748	0.765	0.816	0.894	0.995	0.912	0.843	0.837	0.822	0.871	0.982	0.847	0.660	0.500	0.389	0.423	0.412	0.404

EDD, end-diastolic diameter; GD, gestational diabetes mellitus; LV, left ventricle; RV, right ventricle; Z-AC, Z-score adjusted to the abdominal circumference; Z-EFW, Z-score adjusted to the estimated fetal weight; Z-GA, Z-score adjusted to the gestational age; SD, standard deviation; *p*, *p*-value.

Table S3. Comparison of the 24-segment Sphericity Index and Fractional Shortening between the Control group and the GD group.

24-segments				1	2	3	4	5	6	7	8	9	10	11	12	13	14	15	16	17	18	19	20	21	22	23	24
Sphericity Index	LV	Control	Mean	2.12	2.07	2.02	2	1.99	2.01	2.06	2.12	2.19	2.25	2.32	2.4	2.48	2.58	2.69	2.81	2.92	3.03	3.17	3.4	3.85	4.76	6.8	13.21
			SD	0.33	0.29	0.28	0.27	0.27	0.27	0.27	0.27	0.28	0.29	0.3	0.32	0.34	0.36	0.38	0.4	0.43	0.47	0.52	0.6	0.72	0.93	1.36	2.69
		GD	Mean	2.01	2	1.98	1.98	1.99	2.03	2.08	2.14	2.22	2.29	2.36	2.44	2.53	2.64	2.75	2.86	2.97	3.07	3.19	3.41	3.83	4.73	6.73	13.06
			SD	0.34	0.32	0.31	0.3	0.3	0.31	0.32	0.34	0.36	0.38	0.4	0.42	0.44	0.46	0.48	0.49	0.5	0.51	0.53	0.6	0.72	0.95	1.41	2.8
			p	0.224	0.365	0.587	0.814	0.996	0.832	0.768	0.743	0.708	0.675	0.638	0.605	0.597	0.589	0.595	0.630	0.379	0.372	0.453	0.607	0.932	0.871	0.839	0.820
	RV	Control	Mean	1.74	1.73	1.72	1.71	1.71	1.73	1.75	1.78	1.82	1.87	1.94	2.01	2.09	2.19	2.29	2.39	2.51	2.62	2.77	3.01	3.44	4.28	6.14	11.96
			SD	0.22	0.22	0.22	0.23	0.24	0.24	0.25	0.25	0.26	0.28	0.3	0.32	0.34	0.36	0.39	0.41	0.43	0.44	0.46	0.5	0.59	0.77	1.15	2.29
		GD	Mean	1.75	1.75	1.75	1.75	1.76	1.78	1.8	1.83	1.88	1.92	1.98	2.05	2.12	2.21	2.32	2.44	2.57	2.72	2.92	3.23	3.74	4.7	6.79	13.28
			SD	0.33	0.33	0.32	0.32	0.32	0.33	0.34	0.35	0.37	0.38	0.4	0.42	0.44	0.47	0.5	0.54	0.58	0.62	0.68	0.77	0.92	1.21	1.79	3.55
			p	0.945	0.814	0.669	0.601	0.729	0.807	0.767	0.714	0.787	0.872	0.979	0.933	0.938	0.918	0.953	0.928	0.959	0.807	0.639	0.393	0.310	0.272	0.261	0.245
Sphericity Index (Z-score)	LV	Control	Mean	0.17	0.08	0	-0.07	-0.09	-0.05	0.04	0.16	0.29	0.4	0.49	0.56	0.62	0.68	0.74	0.8	0.88	0.98	1.08	1.16	1.21	1.24	1.25	1.26
			SD	0.79	0.74	0.73	0.74	0.75	0.76	0.76	0.76	0.76	0.76	0.77	0.78	0.79	0.79	0.79	0.79	0.83	0.89	0.97	1.04	1.09	1.12	1.14	1.16
		GD	Mean	-0.06	-0.09	-0.11	-0.12	-0.09	-0.01	0.1	0.24	0.37	0.49	0.59	0.68	0.75	0.81	0.86	0.91	0.97	1.05	1.12	1.17	1.19	1.19	1.19	1.19
			SD	0.82	0.81	0.81	0.82	0.85	0.88	0.91	0.94	0.98	1.01	1.03	1.04	1.04	1.02	0.99	0.97	0.95	0.96	0.99	1.04	1.1	1.15	1.18	1.2
			p	0.226	0.370	0.576	0.813	0.986	0.852	0.778	0.736	0.711	0.676	0.645	0.611	0.596	0.586	0.594	0.622	0.379	0.369	0.460	0.599	0.927	0.870	0.838	0.825
	RV	Control	Mean	-0.17	-0.16	-0.15	-0.12	-0.09	-0.04	0.02	0.09	0.17	0.25	0.32	0.39	0.44	0.47	0.47	0.45	0.44	0.44	0.44	0.43	0.42	0.4	0.39	0.38
			SD	0.67	0.68	0.7	0.74	0.77	0.79	0.8	0.81	0.83	0.85	0.88	0.91	0.93	0.92	0.91	0.89	0.88	0.87	0.86	0.86	0.87	0.89	0.9	0.9
		GD	Mean	-0.16	-0.11	-0.06	0	0.05	0.12	0.19	0.27	0.33	0.39	0.45	0.49	0.52	0.53	0.54	0.55	0.57	0.64	0.72	0.8	0.86	0.88	0.9	0.9
			SD	1	1.02	1.03	1.04	1.06	1.07	1.09	1.12	1.14	1.17	1.18	1.2	1.2	1.19	1.18	1.17	1.18	1.22	1.27	1.32	1.36	1.39	1.4	1.4
			p	0.939	0.813	0.691	0.596	0.733	0.792	0.792	0.686	0.768	0.882	0.989	0.923	0.923	0.928	0.953	0.918	0.964	0.812	0.630	0.400	0.317	0.275	0.259	0.248
Fractional Shortening (%)	LV	Control	Mean	15.16	17.84	20.32	22.57	24.56	26.32	27.95	29.52	31.06	32.57	34.03	35.44	36.82	38.22	39.61	40.94	42.11	43.04	43.66	44.02	44.2	44.29	44.34	44.36
			SD	7.92	7.14	6.96	7.15	7.43	7.66	7.89	8.15	8.4	8.56	8.7	8.89	9.07	9.09	9.01	8.87	8.7	8.55	8.7	9.29	10.12	10.85	11.36	11.66
		GD	Mean	16.24	18.35	20.38	22.32	24.18	25.99	27.76	29.45	30.99	32.3	33.39	34.36	35.31	36.31	37.36	38.38	39.24	39.82	40.13	40.24	40.24	40.2	40.17	40.15
			SD	8.39	7.62	7.59	8.05	8.59	8.95	9.25	9.6	9.92	10.05	10.02	9.93	9.88	9.92	10.15	10.55	10.98	11.33	11.82	12.56	13.44	14.19	14.73	15.04
			p	0.591	0.780	0.974	0.895	0.908	0.817	0.676	0.695	0.976	0.905	0.783	0.644	0.519	0.420	0.345	0.290	0.242	0.197	0.171	0.169	0.180	0.193	0.203	0.208
	RV	Control	Mean	13.03	14.8	16.49	18.05	19.43	20.62	21.63	22.47	23.16	23.7	24.14	24.55	25.06	25.74	26.45	26.98	27.11	26.69	25.84	24.77	23.71	22.91	22.39	22.09
			SD	7.11	6.27	6.09	6.35	6.71	6.97	7.21	7.46	7.7	7.88	8.12	8.47	8.84	9.18	9.73	10.51	11.14	11.17	10.8	10.46	10.48	10.75	11.05	11.26
		GD	Mean	10.84	11.63	12.37	13.03	13.59	14.05	14.43	14.77	15.09	15.4	15.68	15.88	16.02	16.11	16.12	16.04	15.83	15.44	14.85	14.09	13.27	12.6	12.14	11.86
			SD	9	8.35	8.21	8.51	9	9.56	10.3	11.25	12.23	13.02	13.63	14.09	14.3	14.12	13.72	13.35	13.16	13.29	13.92	15.11	16.61	17.92	18.85	19.4
			p	0.277	0.086	0.064	0.008	0.003	0.002	0.001	0.001	0.002	0.004	0.005	0.001	<0.001	<0.001	<0.001	<0.001	<0.001	0.001	0.003	0.006	0.003	0.006	0.030	0.042
Fractional Shortening (Z-score)	LV	Control	Mean	-0.37	-0.12	0.1	0.27	0.42	0.49	0.48	0.41	0.32	0.22	0.14	0.09	0.05	0.04	0.05	0.05	0.04	0	-0.05	-0.12	-0.17	-0.19	-0.21	-0.22
			SD	0.94	0.87	0.87	0.93	0.96	0.97	1.02	1.11	1.16	1.16	1.13	1.09	1.04	0.98	0.92	0.85	0.79	0.73	0.7	0.72	0.74	0.76	0.77	0.78
		GD	Mean	-0.24	-0.06	0.11	0.24	0.37	0.45	0.45	0.4	0.31	0.19	0.06	-0.04	-0.11	-0.16	-0.17	-0.19	-0.22	-0.27	-0.33	-0.42	-0.46	-0.48	-0.49	-0.5
			SD	1	0.93	0.95	1.05	1.11	1.13	1.2	1.3	1.37	1.36	1.3	1.22	1.14	1.08	1.03	1.01	0.99	0.97	0.95	0.98	0.98	0.99	1	1
			p	0.590	0.782	0.973	0.900	0.903	0.817	0.676	0.695	0.975	0.906	0.781	0.642	0.516	0.423	0.345	0.290	0.242	0.196	0.170	0.168	0.180	0.192	0.203	0.209
	RV	Control	Mean	-0.65	-0.52	-0.35	-0.19	-0.06	-0.02	-0.03	-0.06	-0.11	-0.15	-0.18	-0.17	-0.12	-0.03	0.06	0.12	0.12	0.05	-0.03	-0.14	-0.26	-0.37	-0.41	-0.43
			SD	0.84	0.79	0.79	0.85	0.93	1.05	1.15	1.21	1.23	1.2	1.16	1.13	1.1	1.08	1.06	1.08	1.1	1.05	1.05	0.92	0.87	0.79	0.78	0.78
		GD	Mean	-0.91	-0.92	-0.89	-0.86	-0.88	-1.01	-1.18	-1.32	-1.41	-1.42	-1.39	-1.33	-1.25	-1.16	-1.06	-1	-0.99	-0.99	-1.1	-1.08	-1.13	-1.13	-1.15	-1.15
			SD	1.06	1.05	1.07	1.14	1.26	1.44	1.64	1.83	1.96	1.99	1.96	1.88	1.79	1.65	1.5	1.38	1.3	1.24	1.35	1.33	1.38	1.31	1.34	1.35
			p	0.274	0.085	0.064	0.008	0.004	0.002	0.001	0.001	0.002	0.004	0.005	0.003	0.003	0.001	<0.001	<0.001	<0.001	<0.001	<0.001	0.001	0.003	0.006	0.030	0.042

GD, gestational diabetes mellitus; LV, left ventricle; RV, right ventricle; GD, gestational diabetes; SD, standard deviation; *p*, *p*-value.
